# COVID-19 Tracheostomy: Experience in a University Hospital With Intermediate Follow-up

**DOI:** 10.1177/08850666211043436

**Published:** 2021-10-29

**Authors:** Rahul N Sood, Benjamin A. Palleiko, Daniel Alape-Moya, Mark W. Maxfield, Jonathan Holdorf, Karl Fabian Uy

**Affiliations:** 1 UMass Memorial Medical Center, Worcester, MA, USA; 2 12262 University of Massachusetts Medical School, Worcester, MA, USA

**Keywords:** tracheostomy, coronavirus-2019, critical care

## Abstract

The benefits of percutaneous dilational tracheostomy (PDT) placement have been well documented in patients requiring prolonged mechanical ventilation. However, the data regarding the benefit of PDT in coronavirus-2019 (COVID-19) patients are scarce. The objective of this study is to evaluate the outcomes of a cohort of 37 patients who underwent tracheostomy as part of their COVID-19 care. Retrospective data from a series for 37 patients undergoing tracheostomy was collected using chart review. Primary outcomes included 30 and 60 day mortality, weaning rate, and decannulation rate. Secondary outcomes collected included admission demographics, comorbidities, and procedural information. Thirty-seven (37) patients requiring prolonged mechanical ventilation due to COVID-19. Of these 37 patients, 35 were alive 60 days post-PDT placement, 33 have been weaned from mechanical ventilation and 18 have been decannulated. The low mortality and high decannulation rates in this cohort in is a promising development in the care of critically ill COVID-19 patients. Of note, all participating physicians underwent routine polymerase chain reaction (PCR) testing for infection with the severe acute respiratory syndrome coronavirus-2 virus and no physician contracted COVID-19 as a result of their involvement. Overall, this case series describes the modified PDT technique used by our team and discusses the feasibility and potential benefit to PDT placement in COVID-19 patients requiring long-term mechanical ventilation.

## Background

Severe acute respiratory syndrome coronavirus-2 (SARS-CoV-2) was first described in Wuhan, China as early as December, 2019 and has led to a pandemic that has infected more than 120 million people and has taken more than 2.6 million lives.^1^ A recent review suggests that up to 26% of patients hospitalized with coronavirus-2019 (COVID-19) will require admission to the intensive care unit (ICU), and that mortality in from COVID-19 once in the ICU may be as high as 31%.^2^ Management of severe disease secondary to COVID-19 infection is constantly changing, but often requires prolonged intubation due to acute respiratory failure, neuromuscular blockade, and prone positioning.^3^ Percutaneous dilational tracheostomy (PDT) placement has been shown to facilitate mechanical ventilation weaning, minimize sedation requirement and improve tracheobronchial toileting in patients that require prolonged mechanical ventilation. However, the timing for tracheostomy placement and weaning protocols have not been widely agreed upon or extensively documented.^4^ Initial recommendations from the American Academy of Otolaryngology for the timing of COVID-19 tracheostomy placement were to wait a minimum of 14 days after intubation in order to have a better idea of the individual patient prognosis.^5^ Other studies have suggested that the window for a safe tracheostomy is anywhere from 10 to 21 days after intubation.^6^ In a more recent analysis of COVID trach protocols from 26 countries, 91% of protocols that mentioned timing suggested waiting at least 14 days after initiation of mechanical ventilation.^7^ However, recommendations have been made that suggest tracheostomy may be indicated as soon as 7 days after intubation.^4^

Aerosol-generating procedures such as tracheostomy placement are high risk procedures for infection of droplet-transmitted viruses to health care providers.^8^ Nevertheless, the original Angel et al. cohort of 98 COVID-19 patients that underwent PDT demonstrated feasibility and safety of a modified technique that placed bronchoscope alongside the endotracheal tube (ETT) in order to minimalize aerosolization.^9^ Many other studies have also demonstrated the safety and efficacy of the modified PDT technique, but data on patient weaning, decannulation, and mortality is limited, with few studies reporting follow-up duration beyond 30 days.^4^ In addition, mortality rates after tracheostomy are also highly variable, with some centers reporting rates as low as 5%, and others as high as 60%.^4^ More data regarding weaning, decannulation, and mortality along with longer-term follow-up of COVID-19 patients that underwent tracheostomy is necessary in order to optimize patient care for those hospitalized with COVID-19 complications. In this article, we describe a novel modified technique of the Ciaglia dilatational percutaneous tracheostomy, 30 and 60 day mortality, weaning data, and decannulation rates.

## Methods

### Study Design and Patient Selection

This is a single-center case series of 37 patients admitted for COVID-19 who underwent tracheostomy placement at the University of Massachusetts Memorial Medical Center from March 2020 to July 2020. The study received approval from the University of Massachusetts Medical School Institutional Review Board (UMMS IRB). Informed consent was waived by the UMMS IRB due to the retrospective nature of this study. All methods for this study were performed in accordance with the relevant guidelines and regulations put in place by the UMMS IRB.

When tracheostomy placement was deemed necessary by the intensive care team to aid in ventilator weaning for patients with severe COVID-19 pneumonia, the patient was evaluated by our multidisciplinary team for bedside PDT. Patients that were intubated when they had a Glasgow Coma Scale less than or equal to 8, had significant respiratory distress or were hypoxic or hypercapnic with tachypnea greater than 30 breaths per minute. Patients that were candidates for tracheostomy met these criteria for intubation and were anticipated to require long-term mechanical ventilation. Recent recommendations for COVID-19 tracheostomy that include the interruption of ventilation include performing an apnea test prior to the procedure in order to maintain positive end-expiratory pressure (PEEP) in the breathing circuit and minimize the risk of alveolar collapse.^10^ In line with these recommendations, all patients in our cohort underwent an apnea trial prior to tracheostomy placement. If bedside tracheostomy could not be safely performed for anatomical reasons, the patient was taken to the operating room for surgical tracheostomy by thoracic surgery.

### Bedside Tracheostomy Technique

The procedure was performed with a maximum of 4 providers in the room. This included an attending surgeon, bronchoscopist, anesthesiologist, and respiratory therapist (RT). The presence of an anesthesiologist to administer neuromuscular blocking agents for bedside procedures is required per institutional protocol. Providers were required to wear Powered Air-Purifying Respirator (PAPR) along with an N95 mask per our institutional protocol. Medications necessary for procedural sedation and neuromuscular blockade were available prior to time-out. All necessary supplies for the tracheostomy were gathered outside the room along with standby equipment including an intubation box, video laryngoscope, and open tracheostomy kit. At 30 min prior to the start of the procedure, FiO_2_ was increased to 100%, and 15 min prior, the sedative infusion was initiated targeting a Richmond Agitation and Sedation Scale goal of -3. Once the patient was appropriately positioned, the anesthesiologist administered a neuromuscular blocking agent.

Ciaglia method was used in this study. The tracheostomy kit used in our protocol was Ciaglia Blue Rhino percutaneous tracheostomy set (Cook Medical, Bloomington, IN, USA). The bronchoscopist applied a nasal clip and packed the mouth with wet gauze to reduce aerosol generation. After sterile prep and draping, the surgeon made an incision and bluntly dissected. Subsequently, the ETT was pulled back using a disposable bronchoscope (Ambu ascope [Ambu A/S, Ballerup, Denmark]) for guidance, without cuff deflation if possible, until the tip of the ETT was at the level of the cricoid cartilage. The ETT was pulled back without cuff deflation to minimize the risk of aerosol generation and exposure to the physician performing the bronchoscopy. While not possible to confirm, we do believe the ETT cuff remained below the vocal folds (in the sub-glottic space) while the tracheostomy was performed. If cuff deflation was required to move the ETT, only 2 mL of air was removed at a time. After the trachea was accessed, serial dilation were performed with the pencil followed by the blue rhino dilator. Wet gauze was used to cover the incision between these maneuvers. Before removing the dilator, an apneic maneuver was performed on the ventilator by the respiratory therapist to avoid delivering flow across a freshly created stoma. As mentioned previously, all patients prior to tracheostomy underwent an apnea trial. A tracheostomy tube was inserted with a thumb over the opening until the ventilator tubing was connected. We predominantly used #8.0 Shiley or 8.0 Bivona tight to shaft (TTS), but #7.0 Bivona TTS were occasionally placed. Our local institutional practice has been to place # 8.0 inner diameter tracheostomies to allow passage of a therapeutic bronchoscope for suctioning of mucous plugs during the early perioperative period of a tracheostomy. These are typically downsized to a # 7.0 or 6.0 fourteen days after tracheostomy insertion. Following tracheostomy placement, a flexible bronchoscope was passed through the tracheostomy, confirming the position of the tracheostomy tube. The surgeon secured the tube with two sutures and ties. No staff was allowed inside the room for at least one-hour post procedure unless absolutely required.^11^

### Decannulation Protocol

**Figure 1.**
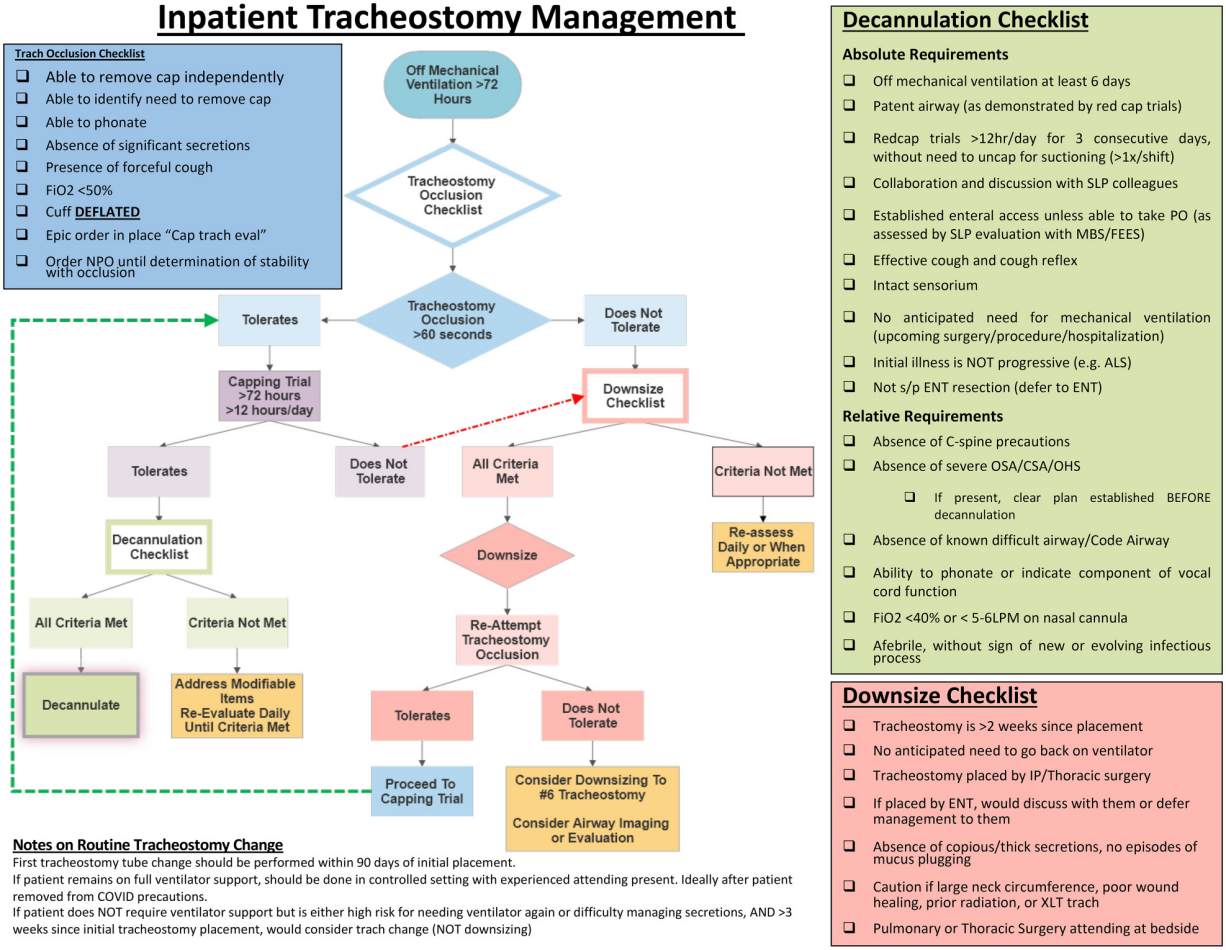
Inpatient tracheostomy management.

After being liberated from mechanical ventilation for 72 h, all patients were started in our decannulation protocol based on multidisciplinary input from services including respiratory therapy, nursing, pulmonology, thoracic surgery, and critical care medicine.^11^ Patients were assessed on a daily basis by our respiratory therapy team. Every patient after being weaned off mechanical ventilation was also evaluated by a speech pathologist for swallowing assessment and speech. In our institution, we make a decision with the RT and speech pathologist regarding which patients are candidates for one-way speaking valves. Speech pathology helps to determine when the patient can advance their diet. Once patients had been weaned off mechanical ventilation for at least 5 days and had a reassuring clinical trajectory including improving sensorium, cough, and swallowing reflexes, they underwent a tracheostomy occlusion trial for 60 s to evaluate upper airway patency. Individuals who tolerated the occlusion trial were candidates for the red cap trail for 12 h/day for three consecutive days. If patients did not develop any respiratory symptoms and met all the items in our decannulation checklist, the tracheostomy tube was removed (without downsizing).^12^ Patients who did not tolerate the occlusion trial were candidates for downsizing after 2 weeks of tracheostomy placement (Figure 1).

### Data Collection and Analysis

Age, gender, and comorbidities were recorded prior to intervention. APACHE II scores were calculated with the APACHE II MDCalc tool (https://www.mdcalc.com/apache-ii-score) from patient lab results at the time of ICU admission. Horowitz Index for Lung Function (P/F Ratio) was also calculated using the MDCalc tool (https://www.mdcalc.com/horowitz-index-lung-function-p-f-ratio). If labs were not obtained immediately at the time of ICU admission, the closest lab values to the admission were used to calculate the APACHE II score. When calculating the P/F ratio, the arterial blood gas (ABG) values closest to the date of tracheostomy were used. The remaining four patients required tracheostomy due to the need for prolonged mechanical ventilation. COVID-19 infection (positive or negative) at the time of tracheostomy was determined by using the result of the SARS-CoV-2 PCR or nucleic acid testing test closest to the date of tracheostomy. Patients admitted for COVID-19 were routinely tested for the presence of the SARS-CoV-2 virus and thus laboratory results were readily available via chart review.

Procedural data regarding the tracheostomy (room into room out time and incision to tracheostomy placement time) was collected at the time of the tracheostomy initially for internal purposes to maximize efficiency and minimize provider time in the room and thus minimize exposure risk. This exception to the retrospective data collection of the study was included in the submission to the institutional review board and was approved for use along with the rest of the study. The last day of data collection was July 12, 2020 and this date was used to calculate the duration of follow-up time. Primary endpoints for the study were 30 and 60 day mortality, discharge disposition, and decannulation rate. Additional data collected included operative data from the tracheostomy placement procedure. Continuous variables were analyzed using mean and standard deviation. Dichotomous variables were analyzed as a percentage.

## Results

### Patients

Of the 37 patients with persistent respiratory failure due to COVID-19 pneumonia that underwent tracheostomy placement, 36 (97%) were done at the bedside. All procedures were done using PDT with our previously described technique.^11^ The mean age was 59 standard deviation (SD = 16) years, 22 (59%) were male, and mean body mass index was 32 (SD = 7.4) (Table 1). The mean APACHE II score at the time of ICU admission was 19 (SD = 8.7). The mean P/F ratio using ABG values closest to tracheostomy was 209 (SD = 86). Of the 37 patients in the cohort, 33 (89%) had a P/F ratio <300. At the time of tracheostomy placement, 23 (62%) patients had positive COVID-19 PCR testing in the past 24 h.

**Table 1.** Demographic and Tracheostomy Information.

Demographics	Patients (*n* = 37)
Age—mean (SD)	59 (16)
BMI—mean (SD)	32 (7.4)
Sex—*n* (%)	
Female	15 (41)
Male	22 (59)
Comorbidities—*n* (%)	
COPD	1 (3)
Diabetes	17 (46)
Obesity	21 (56)
APACHE II score—mean (SD)	19 (8.7)
P/F ratio at closest time to tracheostomy—mean (SD)	209 (86)
P/F ratio <300—*n* (%)	33 (89)
Requiring RRT—*n* (%)	5 (13)
Proning—*n* (%)	31 (84)
Tracheostomy data	
Extubation trial prior—*n* (%)	
Yes	18 (49)
No	19 (51)
COVID-19 PCR status at time of procedure—*n* (%)	
Positive	23 (62)
Negative	14 (38)
Days on mechanical ventilation prior—*n* (range)	22 (15-29)
Type of tracheostomy—*n* (%)	
Shiley	22 (59)
Bivona	15 (41)
Size—*n* (%)	
7 mm	5 (14)
8 mm	32 (86)
Room in—room out in minutes (SD)	28 (6.5)
Incision to tracheostomy placement in minutes (SD)	5 (2)

SD, standard deviation; BMI, body mass index; COVID-19, coronavirus-2019.

Prior to tracheostomy, patients spent a mean of 22 days on mechanical ventilation and 18 (48%) patients had failed a trial of extubation earlier in their ICU course. The time the PDT team spent in the room was 28 (SD = 6.5) minutes. Mean skin incision to tracheostomy placement time was 5 min (SD = 2).

### Intervention Outcomes and Complications

The mean length of mechanical ventilation after PDT was 17 (SD = 15) days. Patients have followed an average of 62 days but due to the retrospective nature of the study and the proximity of data collection to tracheostomy placement, not all patients were followed for the full 60 days. Of these 37 patients, 33 (89%) have successfully been weaned from mechanical ventilation and 18 (48%) have been decannulated with a mean time of 17 (SD = 15) days and 26 (SD = 14) days respectively. At the end of study 31 (88%) patients were discharged alive from the ICU, of which 28 (76%) were discharged to a rehabilitation facility and 7 (19%) discharged home. The proportion of patients weaned off supplemental oxygen was 51% (Table 2).

**Table 2.** Outcomes After Percutaneous Tracheotomy Placement.

Outcomes	Value
Complications—*n* (%)	
Bleeding	4 (11)
Cellulitis	3 (8)
Dislodge	2 (5)
Days on mechanical ventilation after tracheostomy (SD)	17 (15)
Weaned from mechanical ventilation—*n* (%)	
Yes	33 (89)
No	4 (11)
Disposition—*n* (%)	
Home	7 (19)
Acute care rehabilitation	28 (76)
Deceased	2 (5)
Decannulated—*n* (%)	18 (49)
Days to decannulation (SD)	26 (14)
Weaned from supplemental O_2_—*n* (%)	19 (51)
Alive at 30 day posttrach—*n* (%)	35 (95)
Alive at 60 day posttrach—*n* (%)	35 (95)
Duration of follow-up	62 (16)

SD, standard deviation.

Two patients died during the follow-up time; one was of multiorgan failure from ventilator-associated pneumonia 14 days post-PDT and the other from multisystem organ failure 55 days post-PDT. Post tracheostomy bleeding requiring the packing of the stoma occurred in 4 (11%) patients, while 2 (5%) patients had accidental tracheostomy removals, one by a patient and another by a provider. Cellulitis of the stoma requiring antibiotics happened in 3 (8%) patients. No patient required reinsertion of their tracheostomy (Table 2).

### Exposure to Personnel

All physicians were tested for COVID-19 by PCR testing at intermittent timepoints between April and July 2020 and all of those tests were negative. All participating physicians had negative nasopharyngeal SARS-CoV-2 PCR tests within 3 weeks of performing a tracheostomy. No physician missed days from work as a result of illness during this period.

## Discussion

Tracheostomy placement has been shown to facilitate weaning for mechanical ventilation, shorten ICU stays, decrease patient anxiety, improve secretion management, and reduce the need for sedation.^13^ Case series data early in the pandemic reported high mortality rates of 50% to 67% for patients with COVID-19 requiring mechanical ventilation. Though our series did not evaluate this specific question, we noted significantly lower mortality rates in our cohort of patients, with a mortality rate of 5%, ventilator weaning rate of 89%, and a decannulation rate of 48%, which is similar to or better than other published case-series data. The original Angel et al. cohort published a 19% weaning rate, 8% decannulation rate, and a 7% mortality rate.^9^ A recent case series analysis from Kwak et al. published a mortality rate of 20%, weaning rate of 73%, and a decannulation rate of 64%.^14^ However, differences in tracheostomy timing and follow-up duration may be contributing to these discrepancies in outcomes.

In our study, we demonstrate a high weaning rate at 89%, with a comparatively low decannulation rate (48%). We believe that this may have to do with the limited duration of follow-up and suspect that given more time the decannulation rate may improve. Additionally, particularly in the later tracheostomy procedures, many of the patients were transferred to outside rehabilitation facilities in order to maximize the number of available inpatient beds. Thus access to their charts was limited depending on the facility to which they were transferred. It is possible that some of the patients who were decannulated at an outside facility were missed during the chart review process. Also of note is patients that who were not decannulated did not undergo laryngoscopic or bronchoscopic evaluation for vocal cord dysfunction, granulation tissue, or stenosis/malacia before the end of the study period. These are all also factors that could be affecting decannulation rates in our study that we are unable to comment on given the data available to us for this cohort. In order to increase the decannulation rate in the future, it may be worthwhile to consider downsizing the tracheostomy tubes prior to the capping trials.

In our cohort, there were relatively high rates of bleeding, accidental decannulation, and stomal infections. It is possible that the high rate of bleeding seen in our patients can be attributed to the high-dose prophylactic anticoagulation that many of our patients were on, particularly early on in the pandemic. However, recent data suggest that high-dose anticoagulation does not lead to a decrease in thromboembolic events in COVID-19 patients when compared to normal dose anticoagulation.^15^ The high rate of accidental decannulation may be due to the high degree of altered mental status and COVID-19 related encephalopathy that we observed in our patient cohort. All but one of the accidental decannulations were due to self-decannulation. We also observed a relatively high rate of infection, which we believe may be due to inexperience with the handling of tracheostomy equipment with the personal protective equipment (PPE) that was required by our institution. Additionally, many of our patients had prolonged hospital stays which may have put them at a higher risk for infection.

A relatively young patient population with a mean age of 59, along with strict adherence to our decannulation algorithm may explain our success in the patient cohort. Decannulation is a key step toward COVID-19 recovery and multiple protocols have been established to accomplish this goal. Our protocol follows recommendations described by previous studies that based their success in daily evaluation, occlusion trials, and downsizing.^16,17^ Significant delays in discharging patients to long-term skilled nursing facilities from our institution also prolonged inpatient hospital stays, which gave our team a chance to initiate the decannulation process in-house. However, later on in the pandemic, many patients were decannulated at rehabilitation facilities. This may have helped to optimize the delivery of care, and initiation of decannulation prior to discharge to a skilled nursing facility may be one approach to improve outcomes following tracheostomy placement for COVID-19 complications.

As mentioned previously, our protocol followed several recommendations described in the literature for tracheostomy evaluation, occlusion trials, and downsizing.^16,17^ It seems that following these recommendations does not increase the risk of infections in health care workers and our study also implemented modifications to the conventional PDT approach in order to reduce aerosolization.^12,18^ These included paralysis for all procedures and interruption of ventilation when the stoma was created. Our institutional protocol mandated the use of a PAPR along with an N95 mask for all personnel in the room, though further studies will need to be done to show if the use of both devices is better than either one. No physicians on the team developed any symptoms suggestive of COVID-19 infection as a result of these procedures. This is a promising and original finding of our study, as to our knowledge there has not been a case series reported in which the physicians performing the tracheostomies were routine tested for COVID-19 infection.

The main strengths of our study include longer duration of follow-up compared to prior series, and adherence to an algorithm to aid in decannulation. Additionally, we demonstrated a high success rate with the safe patient and provider outcomes. Weaknesses include its retrospective nature and relatively small, single-center cohort. Also, we were unable to test other healthcare workers involved in the postprocedural period including RT's and nursing staff. Further studies defining the risk of infection to healthcare workers following tracheostomy placement are needed.

As the COVID-19 pandemic continues, tracheostomy placement may become an important step in weaning patients off mechanical ventilation, thereby expediting recovery and increasing the number of ICU beds. Our technique is a minor variation of the well-described PDT, with modifications to reduce aerosolization. Promising outcomes were noted in this cohort of patients including low mortality and high rates of ventilator weaning and decannulation. Larger studies are needed to further define the long-term outcomes in patients with COVID-19 that undergo tracheostomy placement.
